# Injection sclerotherapy for haemorrhoids using an injection needle via an endoscope

**DOI:** 10.1308/rcsann.2026.0033

**Published:** 2026-08-17

**Authors:** P Batra, A Sharma, A Sharma

**Affiliations:** ^1^ Manchester University NHS Foundation Trust, UK; ^2^ Imperial College, UK; ^3^ University of Manchester, UK

Injection sclerotherapy with 5% oily phenol (Figure 1) is a well-established and effective treatment for haemorrhoids, particularly in patients receiving anticoagulation in whom rubber band ligation is unsuitable.^1^ Previous work has demonstrated the safety and efficacy of this approach.^2^ The unavailability of the proctoscopic haemorrhoid injection needle has created a practical challenge.

This technical note describes an alternative technique using a standard endoscopic injection needle passed through a gastroscope or colonoscope. The procedure is performed as a day case.^3^ The endoscope is introduced and retroflexed (Figure 2) in the rectum to allow clear visualisation of haemorrhoids.

Injection is performed using either a 21-gauge glue-injection needle (Figure 3) via a gastroscope or a 19-gauge injection needle (Figure 4) via a colonoscope. Five per cent oily phenol is drawn into a Luer-lock syringe, and the needle is primed before use. Under direct vision, 1–2ml of sclerosant is injected into the submucosal plane at or just above each haemorrhoidal cushion (total 5–7ml), with aspiration prior to injection to avoid intravascular delivery. Mucosal lifting and blanching confirms correct placement.

Injection sclerotherapy with this technique is safer than the use of proctoscopic needles. Proctoscopic needles are around 9cm long and there is a recognised risk of penetration of adjacent organs, including injection in the prostate leading to prostatitis. The endoscopic needle is longer to enable it to go through the scope, but the active sharp needle is only 3–6mm long, which makes them safer with a negligible risk of penetration through the rectal wall into any adjacent organs.

**Figure 1:**
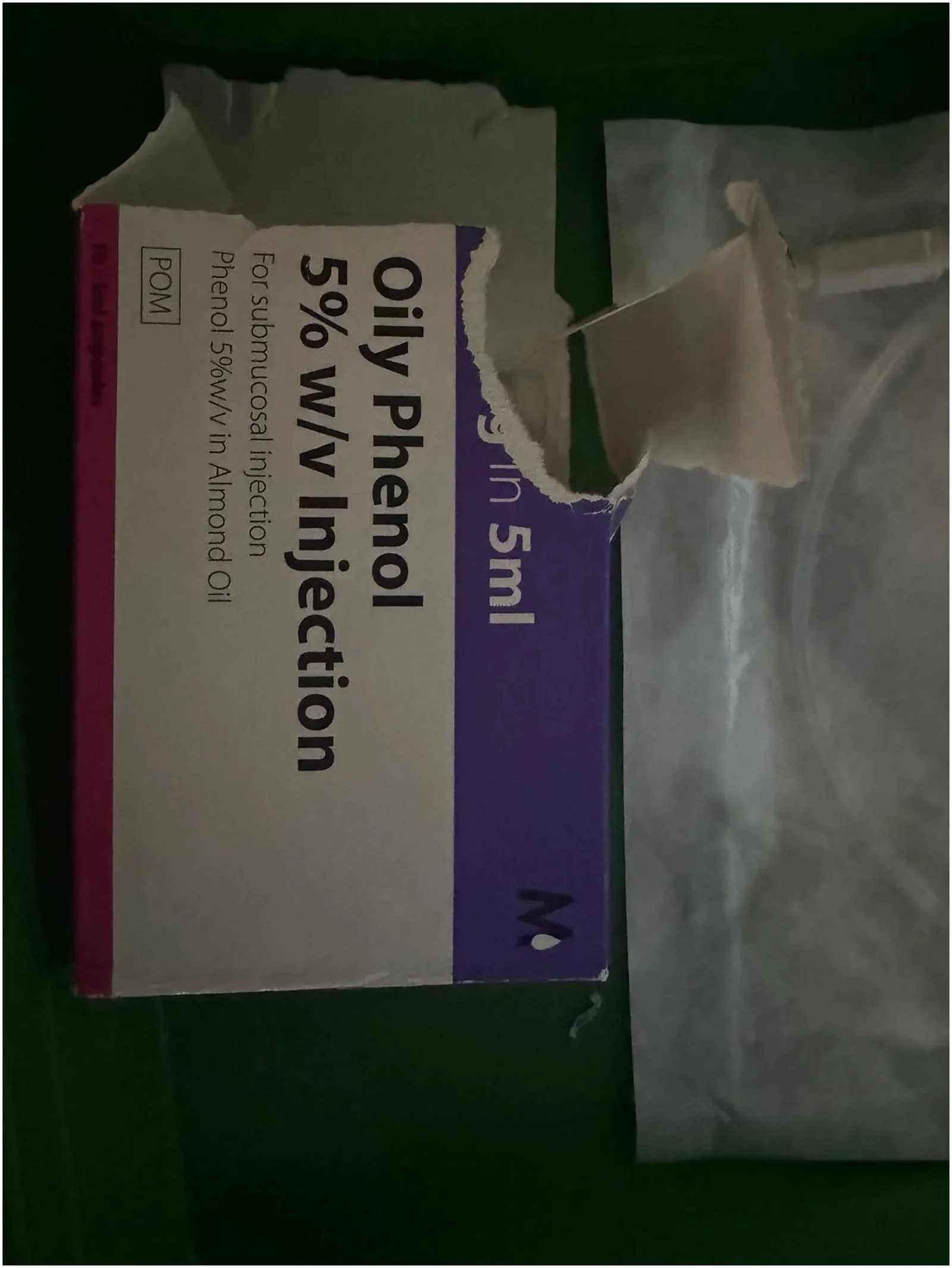
Sclerosant 5% oily phenol

**Figure 2:**
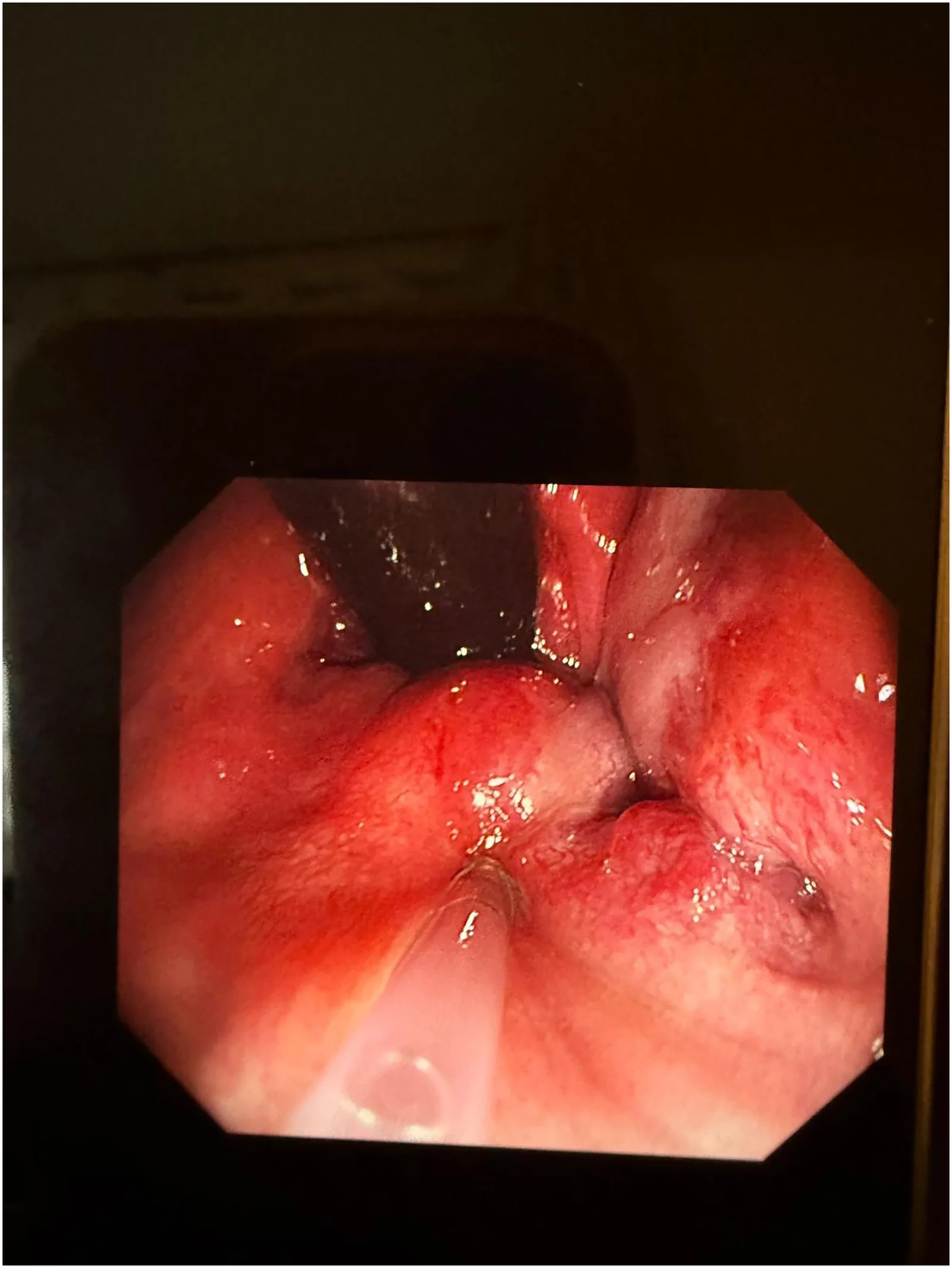
Retroflexed gastroscope view of haemorrhoids

**Figure 3:**
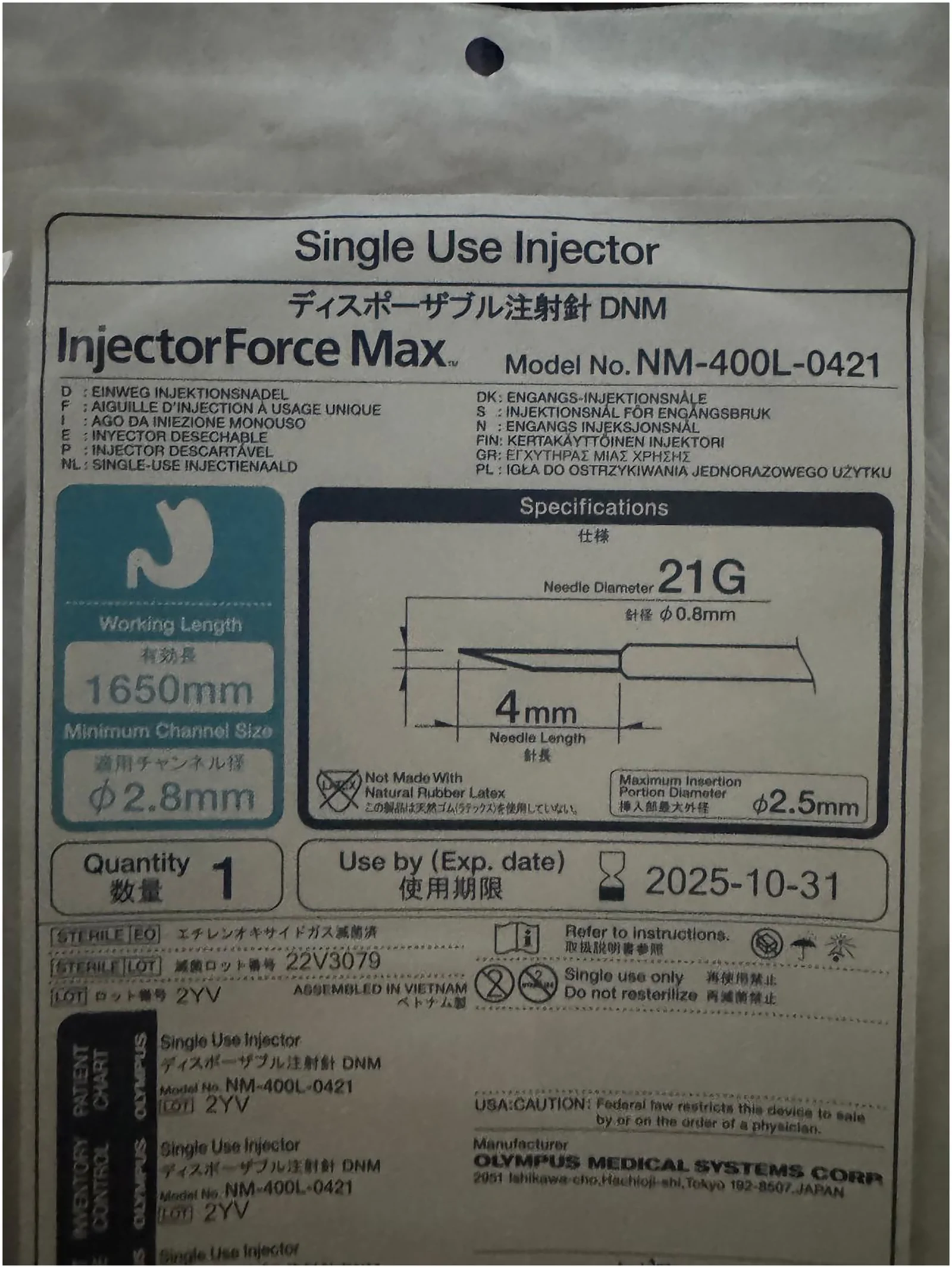
Glue injection needle 21 G InjectorForce Max, OLYMPUS

**Figure 4:**
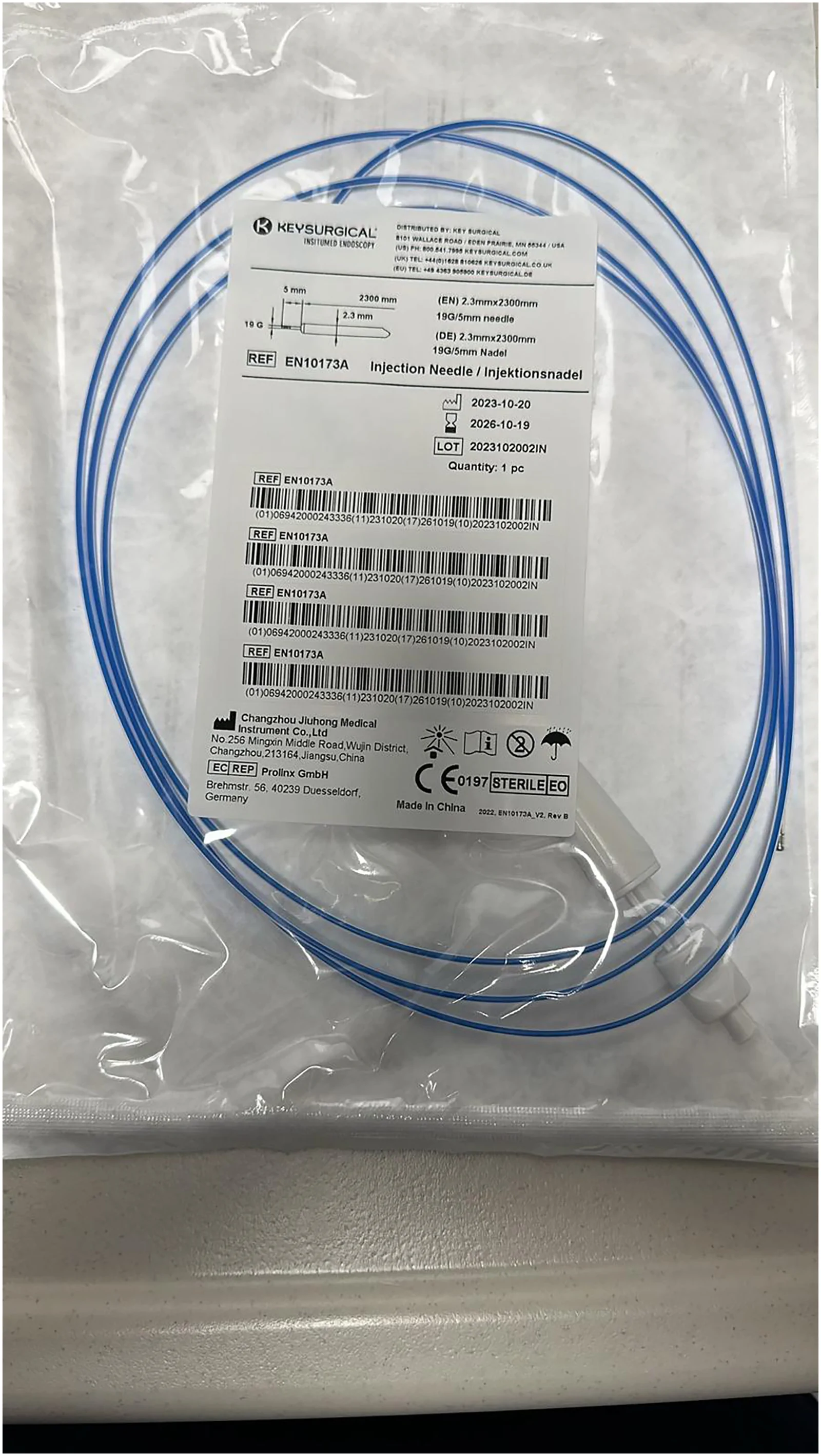
19-gauge needle, KEYSURGICAL

## Competing interests

The authors declare no competing interests.

## Funding

The authors received no financial support for the research, authorship and/or publication of this article.

## Ethics approval

Not required.

## Consent to participate

Consent was obtained prior to procedures but no patient data has been used in this article.

## Author contributions

**P Batra**: Conceptualisation, Project administration, Writing – original draft. **A Sharma**: Conceptualisation, Methodology, Writing – review & editing. **A Sharma**: Conceptualisation, Writing – original draft.

## Artificial Intelligence

The author/s declare that no AI was used to conduct the study or prepare the manuscript.
